# Cross-Layer BLE Device-Type Identification via Locality-Sensitive Hashing for IoT Security

**DOI:** 10.3390/s26217017

**Published:** 2026-11-06

**Authors:** Chaimae Saadouni, Saad El Jaouhari, Nouredine Tamani, Lina Mroueh, Soumia Ziti

**Affiliations:** 1 EDITE Doctoral School, Sorbonne University, 75012 Paris, France; 2 LISITE, Institut Superieur d’Electronique de Paris (Isep), 92130 Issy-les-Moulineaux, France; saad.el-jaouhari@isep.fr (S.E.J.); nouredine.tamani@isep.fr (N.T.); lina.mroueh@isep.fr (L.M.); 3 Faculty of Sciences, Mohammed V University, Rabat 10000, Morocco; s.ziti@um5r.ac.ma

**Keywords:** Bluetooth Low Energy, BLE, device fingerprinting, device-type identification, Internet of Things, security, locality-sensitive hashing, Nilsimsa

## Abstract

Bluetooth Low Energy (BLE) devices are ubiquitous across consumer, industrial, and healthcare IoT ecosystems, yet their resource-constrained nature typically precludes conventional cryptographic authentication, leaving device identification as a security primitive for device management, access control, and threat detection in IoT environments. Current BLE fingerprinting techniques are restricted by rigid cryptographic hashes, machine learning models that require periodic retraining to cover new devices, or single-layer feature sets vulnerable to missing data. In response to these limitations, we introduce a Nilsimsa-based identification approach that performs BLE device type/manufacturer identification through merging link-layer features and advertised GATT service information before network access is granted to cope with missing or inaccessible data. We analyzed various combinations of features from different layers to assess how merging them impacts identification accuracy. The exhaustive analysis uses 10 test objects and 10 reference hashes. The large-scale evaluation uses 5000 test records and 10,000 reference hashes covering 104 device type–manufacturer pairs. Key findings highlight that cross-layer features are important to cope with missing features. The minimum subset size was found at k = 10 with a subset-coverage criterion: at least 95% of all subsets of 10 features reach an accuracy of 90% or more. Our proposal not only facilitates the seamless enrollment of new devices through reference-hash registration without retraining but also enables confidence-based anomaly detection using its similarity score, providing an incrementally extensible approach for BLE security monitoring. Overall, our approach advances BLE fingerprinting by introducing a schema-agnostic, retraining-free, cross-layer identification model tailored for the dynamic environment of IoT deployments.

## 1. Introduction

Widespread interconnection of devices and continuous technological progress, enabled by instant data availability, have paved the way for innovative applications and services for the end-users in a variety of sectors and industries, ranging from autonomous vehicles that interact within urban networks to medical technology that provides patient self-monitoring from any location. BLE has emerged as a key connectivity technology for IoT applications because of its low cost, ease of implementation, and energy efficiency. However, the features that contribute to its rapid adoption also contribute to expanding the vulnerability of interconnected environments [1]. Moreover, BLE’s simplified protocol architecture coupled with its insecure or weak pairing mechanisms, absence of centralized security management, and prevalent implementation or configuration flaws, render devices susceptible to various threats such as eavesdropping, man-in-the-middle attacks, denial of service, unauthorized access, privacy breaches, and device tracking [1,2]. While the Bluetooth specification integrates security protocols to mitigate many of these risks, such as diverse device pairing mechanisms, optional encryption and authentication of connections, and address randomization, the efficacy of these safeguards hinges on their accurate specification, utilization, and implementation [3]. Notably, several devices still rely on vulnerable “Just Works” pairing methods or lack robust application-layer authentication protocols, underscoring persisting security gaps in BLE technology [4]. These challenges are further compounded by the severe memory, computational, energy, and physical-size constraints of many IoT and BLE devices, which can make conventional cryptographic mechanisms costly or impractical to implement [5].

Existing IoT device-identification techniques remain constrained by the limitations of explicit identifiers and the operational costs associated with intrusive profiling [6]. Conventional identification methods such as IP and MAC addresses are not ideal due to their susceptibility to spoofing, susceptibility to alteration through software, vulnerability to mobility-related issues, and the increasing use of randomization techniques that, while intended to enhance privacy, often do not effectively prevent re-identification or tracking in various operational scenarios. Active probing can provide more detailed fingerprints in certain scenarios, but it necessitates bidirectional communication with the target device, potentially increasing communication and computational overhead, disrupting normal operation, and limiting applicability to resource-constrained IoT devices [7]. These constraints have encouraged the adoption of passive fingerprinting techniques that infer identity from observable network activities without directly engaging with the device, thus ensuring seamless operation, minimal impact on resource-constrained nodes and robust identification capabilities across varied deployments. Device fingerprinting refers to the process of determining the specific type and model of IoT devices based on their distinctive network traffic patterns, whereas event or activity detection focuses on identifying specific actions, such as switching on a light, adjusting a thermostat, or streaming audio. Accurate device identification is therefore essential for network administration and privacy protection because it enables operators to identify the type and manufacturer of newly connected devices, configure appropriate firewall policies, detect unpatched vulnerabilities, and update intrusion-detection rules. This capability is important because devices from the same manufacturer may share common vulnerabilities and receive coordinated firmware updates, meaning that a single flaw can affect multiple products from the same vendor [8].

In this regard, accurate and efficient device identification remains a significant challenge due to the intrinsic resource limitations of IoT devices, and their energy-saving strategies, including periodic sleep modes, minimal data transmissions, and lightweight computational operations. This communication behavior substantially limits the amount and diversity of information available for device identification, and relying on restricted feature sets increases the risk of misclassifications. Feature engineering therefore constitutes a critical stage in building effective identification systems, with judicious feature selection playing a decisive role in enhancing both the performance and accuracy of IoT device type-manufacturer identification [9]. The diversity of manufacturers, hardware components, and communication protocols (such as BLE, Zigbee, and Wi-Fi) renders any identification system trained on fixed features progressively obsolete. To address this challenge, a framework is needed that offers incremental scalability and protocol extensibility, accommodates new BLE devices without complex feature engineering or preprocessing, and adapts to newly deployed devices without retraining [10].

A persistent deployment challenge in prior IoT fingerprinting studies is the limited exploration of real-world operating conditions, including incomplete feature availability, heterogeneous device populations, and large-scale identification workloads. These works assess their methods under controlled conditions and often assume that the complete set of GATT or broadcast features is consistently available during inference. In practice, a deployed fingerprinting system must cope with missing or partially observable data, whether due to devices withholding certain GATT characteristics, connection failures preventing feature extraction, or manufacturers restricting fields for privacy reasons. Existing studies do not systematically test how their solutions degrade when only a subset of features can be collected, nor do they validate their systems across a representative fleet of diverse devices. Therefore, we introduce a similarity-based device identification approach built upon the Nilsimsa locality-sensitive hashing scheme.

Nilsimsa is a locality-sensitive hashing technique that offers two advantages: (i) it is schema-agnostic, operating directly on raw byte sequences, which allows structured Generic Attribute Profile (GATT) and unstructured (broadcast) data to be aggregated without normalization, exhaustive feature engineering, or schema alignment across varied input formats, and (ii) it eliminates the need for model training, since new devices are integrated seamlessly by generating their fingerprints and storing them directly in the reference database.

To the best of our knowledge, this is the first study to systematically combine flow-based and packet-based BLE features across three distinct categories: (i) advertisement pattern features capturing temporal and behavioral transmission characteristics, (ii) broadcast packet features extracted from both ADV_IND and SCAN_RSP frames, and (iii) GATT profile structural features. This comprehensive exploration ensures that the resulting identification system is robust under partial observability, as low-power IoT devices that omit certain attributes to conserve battery life can still be reliably identified.

We evaluated our approach on an exhaustive combination of BLE features, simulating realistic subsets of BLE profiles from various device types and manufacturers, thereby exposing the system to real-world deployment challenges. To evaluate the system’s efficiency in identifying device types and manufacturers, we scaled the evaluation to 10,000 reference entries and 5000 test sets. The minimum subset size was found at k = 10 with a subset-coverage criterion: at least 95% of all subsets of 10 features reach an accuracy of 90% or more. These experiments collectively underscore the effectiveness of our solution, showing that it performs well not only in controlled settings but also in deployment-centric scenarios where the complete feature set assumed by many existing methodologies may not be available.

The remainder of this paper is organized as follows. Section 2 reviews related work. Section 3 presents the proposed identification system, beginning with an overview of the methodology in Section 3.1 and the feature extraction in Section 3.2. Section 4 describes the test setup and experimental design. Section 5 presents the results and discussion, and Section 6 concludes the paper.

## 2. State of the Art

Device identification in BLE networks is still an emerging research area despite the rapid proliferation of BLE-enabled devices across consumer, industrial, healthcare [11], and smart-city IoT ecosystems. Current methodologies leverage features from different layers of the BLE stack, including the link layer and the GATT layer. In what follows, we focus on the two main paradigms for BLE device type identification, namely (i) machine learning and deep learning (ML/DL)-based approaches and (ii) GATT-based fingerprinting. Although ML and DL models have demonstrated high accuracy in identifying IoT devices, their implementation remains challenging. They require retraining or fine-tuning to distinguish unseen device classes, and maintaining their performance is computationally expensive and operationally impractical in large-scale, dynamic IoT deployments. In addition, because IoT devices differ widely in hardware processing capabilities and use diverse communication protocols, a method that scales easily as new devices join the network is necessary. ML and DL models suffer from limited scalability because they demand extensive feature extraction and normalization. Furthermore, they rely on large datasets to reach high accuracy, which often requires considerable processing resources.

GATT-based fingerprinting achieves high-entropy identification by leveraging the rich hierarchical structure of services and characteristics, including system IDs, serial numbers, and manufacturer metadata. These features yield stable and highly discriminative signatures that are frequently accessible without authentication. In ref. [12], the authors introduced BLENDER, a system for discovering and fingerprinting BLE devices using four strategies. Two passive/active scanning methods (STG1 and STG2) identify device MAC addresses, while the other two (STG3 and STG4) generate device fingerprints. Fingerprinting is accomplished by concatenating recovered identifiers, either GATT attributes such as device name, manufacturer, model, and revision (STG3) or the raw MAC address itself (STG4), and hashing the resulting string with SHA-1 to generate a unique fingerprint. Celosia et al. [13] explored the potential of BLE device fingerprinting using GATT profiles. They demonstrated that data drawn from the GATT layer could significantly enhance user tracking capabilities. Using a real-world dataset of over 13,000 GATT profiles collected over five months, they built device fingerprints from artifacts such as service/characteristic UUIDs, handles, properties, and values, then quantified their identifying power using empirical entropy and anonymity-set analysis. In ref. [14], the authors presented BF-IoT, a cross-layer secure identification framework for BLE-based networks that combines network flow features from the link layer (advertising patterns, interval timing) with session-level features from the ATT/GATT service layer (packet sequences, response times). A Random Forest classifier was trained on these feature vectors to distinguish between authorized and potentially spoofed devices. The system was evaluated on a dozen commodity BLE devices and achieved near-perfect authentication accuracy. The authors of [15] introduced a link-layer broadcast fingerprinting approach to classify both the type and vendor of BLE transmitters, using broadcast packets captured via a software-defined radio receiver on the Bluetooth advertising channel. The features were collected from BLE link-layer broadcast packets, notably ADV_IND (connectable) and ADV_NONCONN_IND (non-connectable), which are periodically transmitted in plaintext on advertising channels before authentication and pairing process. After removing explicit identifiers such as MAC addresses, device names, and member UUIDs, the remaining features were encoded into a 200-dimensional feature vector and fed into a Multi-Layer Perceptron (MLP). The resulting classifier achieved 99.8% identification accuracy across 15 BLE device categories from various vendors. Zuo et al. [16] presented BleScope, an automatic method for fingerprinting BLE devices using static UUIDs collected from companion mobile applications. They used backward slicing and value-set analysis (VSA) to extract both hard-coded and dynamically computed UUIDs from Android APKs, reconstructing service-characteristic hierarchies for device identification.

As illustrated in Table 1, a review of existing BLE device identification approaches reveals a common set of limitations. BLENDER [12] eliminates the need for periodic retraining, yet its identification mechanism is based primarily on MAC addresses, which are trivially spoofed, and on specific GATT service attributes that may be absent in minimalist or proprietary device profiles, which restricts its applicability across heterogeneous IoT deployments. Moreover, BLENDER’s use of the SHA-1 cryptographic hash introduces fragility, as even minor changes in device attributes or firmware updates produce entirely different digests, breaking established device identities. Although the study by Celosia et al. [13] provides a comprehensive analysis of the high-entropy attributes embedded within the GATT hierarchy, it remains a theoretical contribution that neither extends to a deployable identification system nor provides experimental evaluation under real-world network conditions. While ML/DL-based BLE fingerprinting systems [14,15] demonstrate high classification accuracy within their original training distributions, they suffer to maintain performance when encountering previously unseen device categories, firmware revisions, or protocol implementations. Addressing this requires retraining, which is resource-intensive: it involves not only acquiring labeled traffic but also updating feature-extraction pipelines, recalibrating normalization parameters, re-validating hyperparameters, and redeploying the full model. BleScope [16] relies entirely on the existence of publicly available companion mobile applications; devices that lack an associated app, or whose apps have been removed from Google Play, remain undetectable. Furthermore, its methodology lacks cross-protocol generalization, as it is specifically engineered to exploit unencrypted service UUIDs.

Motivated by these gaps, in the following sections we detail our device identification approach, which is based on the Nilsimsa locality-sensitive hashing scheme. Unlike cryptographic hashes, Nilsimsa produces proportionally small similarity score changes in response to minor data perturbations caused by measurement noise, MAC address randomization, or slight firmware modifications, thereby avoiding the substantial hash divergence exhibited by the SHA-1-based scheme used in BLENDER. In contrast to ML/DL models, the Nilsimsa algorithm hashes raw byte sequences directly, including structured and unstructured features, into a unified representation without complex feature extraction or normalization, enabling training-free enrollment of unseen device type-manufacturers. Unlike prior work that either stops at theoretical entropy analysis or restricts feature extraction to a single packet type or feature category, we provide a comprehensive study of the joint behavior of broadcast packet features and GATT profiles, in both isolated and combined configurations. This allows us to investigate how missing or partially available features affect identification performance across the full diversity of real-world BLE device type-manufacturers.

## 3. Identification System: A Locality-Sensitive Similarity Approach

In this section, we describe the rationale behind our methodology in Section 3.1. Then, we detail the BLE feature extraction process in Section 3.2.

### 3.1. Proposed Methodology

We present a similarity-based methodology for identifying BLE IoT devices during the pre-association phase, in order to reduce potential attack vectors that could compromise the entire network. In the context of device fingerprinting, a similarity-based method collects distinctive device features, such as hardware, software, or network characteristics, and employs a similarity score metric to quantify how closely two sets of BLE features match. Device identification is then achieved by comparing a new fingerprint with those stored in a database and declaring a match when the similarity exceeds a predefined threshold. Cosine similarity is a widely used metric for vector comparison, although alternative similarity measures may be applied depending on the nature of the features used [15].

In this regard, we propose a Nilsimsa hash-based approach for device identification without requiring extensive feature extraction or normalization. Unlike traditional cryptographic hash functions, which are designed to generate high-entropy outputs such that even minor input variations produce completely different hashes, locality-sensitive hash (LSH) functions, such as Nilsimsa, generate similar hash values for similar inputs [17]. More recently, Nilsimsa was used for traffic classification of Wi-Fi devices [18]. It works by employing a sliding window of five bytes that moves one byte at a time across the input. For each window, it generates trigrams and feeds them to the Tran53 hash function, which outputs an integer i between 0 and 255. The i-th counter in the accumulator, an array of 256 integer counters, is then incremented by one. After processing the entire input, the accumulator holds the frequency counts of all detected trigrams. To generate the final 256-bit Nilsimsa digest, the average of all accumulator values is computed, and each bit position is assigned as follows: if the i-th counter exceeds the average, the corresponding i-th bit is set to 1; otherwise, it is set to 0. The resulting hash is 32 bytes long, and the similarity score between two hashes ranges from −128 (complete dissimilarity) to 128 (complete identity). This score is calculated by counting the number of matching bits between the two hashes and then subtracting 128 from that count.

**Figure 1:**
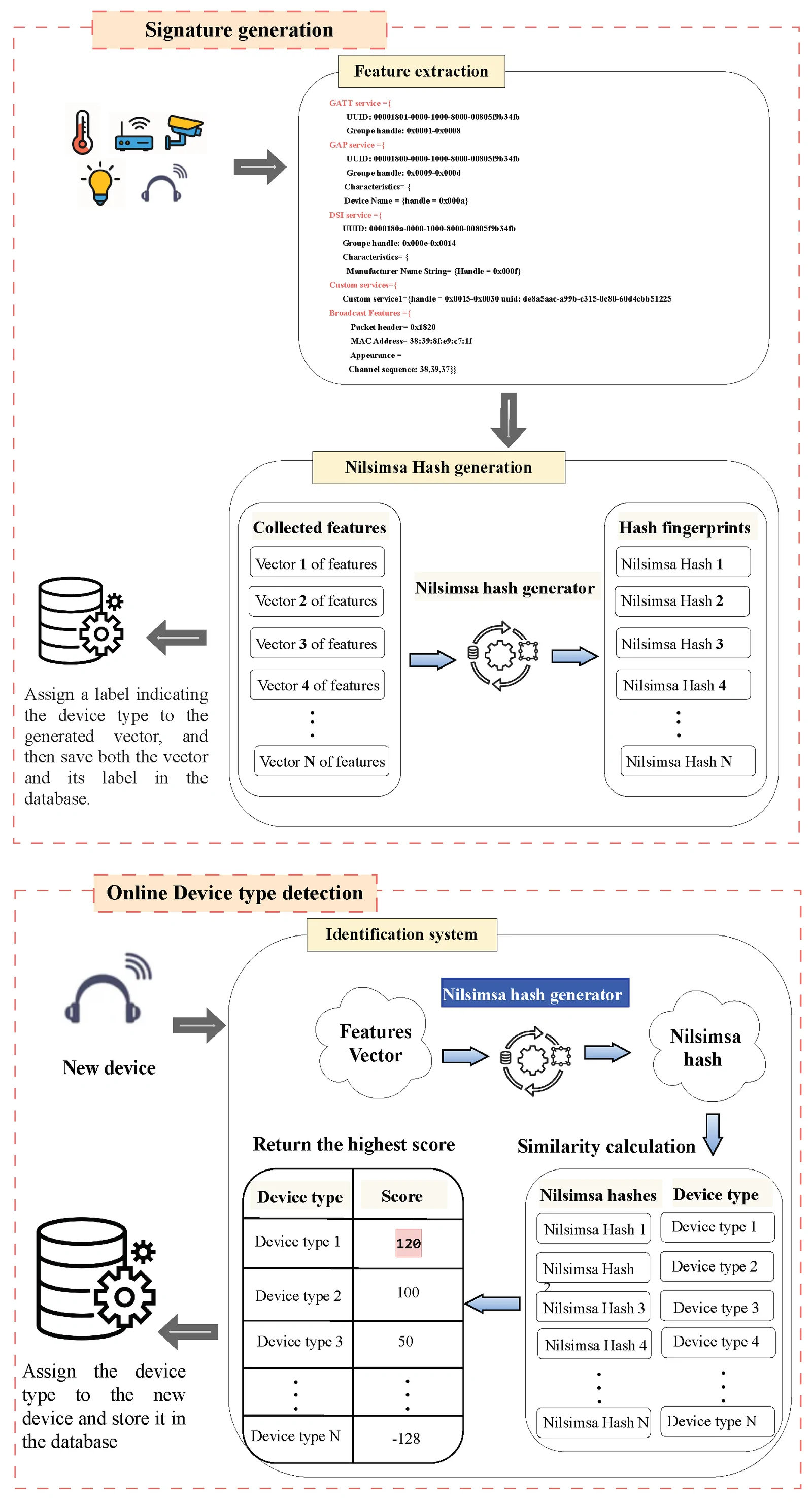
An overview of the device type-manufacturer identification system.

As illustrated in Figure 1, we adopt a two-stage architecture consisting of an offline signature generation phase followed by an online device type-manufacturer detection phase. During the offline stage, a comprehensive set of cross-layer BLE features is passively collected from known legitimate devices, without requiring pairing or authentication. These features encompass link-layer broadcast metadata (advertisement patterns), broadcast packet fields from ADV_IND and SCAN_RSP frames, and hierarchical service-layer attributes such as those of the GATT, Generic Access Profile (GAP), and Device Information Service (DIS) services. The collected information is then organized into a structured JSON profile consisting of five feature categories: broadcast packet attributes, GATT service, GAP service, DIS service, and custom service entries. The extracted features undergo a minimal preprocessing step that only removes null entries and unresolvable attributes, specifically those returning an “Attribute cannot be read” response, thereby retaining the inherent structure of the BLE data without normalization or complex feature engineering. The cleaned feature values are then concatenated into a single string with a fixed procedure. Consecutive values are joined by a single space, numerical and hexadecimal values are kept as text as stored in the JSON, repeated values are kept in their order of appearance. The string is then passed through the Nilsimsa hash generator, which produces a 256-bit locality-sensitive digest for each device record. This digest, along with a label encoding the device type and manufacturer, is stored in a reference hash database, the enrolled fingerprint repository against which future identification queries are resolved. Before hashing, the cleaned JSON profile is converted into one string with a fixed procedure. When a feature subset is evaluated, the selected features keep this same relative order. Furthermore, consecutive values are joined by a single space, and the string is encoded in UTF-8 before it is given to the Nilsimsa function. Numerical and hexadecimal values are kept as text, exactly as stored in the JSON. Null values and attributes that cannot be read are removed during cleaning, so a missing attribute adds nothing to the string and no placeholder is used. Finally, repeated values are kept in their order of appearance, because Nilsimsa counts how many times each trigram occurs. Since Nilsimsa processes the string with a sliding window of five bytes, a change of delimiter or of the order of two fields only modifies the trigrams around the field boundaries, so its effect on the digest is limited. All these choices are nevertheless fixed in every experiment, so that the digests can be reproduced.

In the online identification stage, newly observed devices and previously enrolled devices that reconnect undergo the same feature extraction, preprocessing, and hashing pipeline used during enrollment. Each device’s Nilsimsa digest is compared with all enrolled hashes using the Nilsimsa similarity score, which measures the bit-level similarity between two 256-bit digests on a scale from −128 (maximal dissimilarity) to +128 (a perfect bit-for-bit match). The enrolled reference hash with the highest similarity score relative to the query digest is selected as the closest match, and its associated device type-manufacturer label is assigned to the queried device as the identification result. This assignment is then stored in the database alongside the query fingerprint, allowing the system to maintain a continuously updated registry of observed devices. This registry serves as the foundation for anomaly detection and periodic behavioral re-verification, enabling the gateway to identify deviations from previously established fingerprint baselines across successive monitoring cycles. If the best-matching similarity score falls below a predefined confidence threshold, the device is classified as unidentified and directed to a manual enrollment procedure. This two-phase architecture confines processing to lightweight operations, namely feature extraction, Nilsimsa hash generation, and hash comparison, which makes it well suited to online identification on resource-constrained edge devices.

### 3.2. Multi-Layer BLE Feature Extraction

As mentioned in the previous subsection, we intend to identify IoT devices before they connect to the network. To achieve this, we capture as many features as possible during the pre-association period of a BLE device that does not require pairing, bonding, or authentication. BLE, introduced with Bluetooth 4.0, is a wireless communication technology. BLE is not simply an enhanced version of traditional Bluetooth but a distinct protocol specifically engineered for resource-constrained IoT devices [19], with different design principles and application domains [1]. To establish a connection, the peripheral device periodically broadcasts advertising packets while waiting for a connection request, and the central device scans for advertising packets and initiates the connection. Once established, the devices engage in connection events, which are scheduled intervals in which data is exchanged. Between these events, devices conserve energy by entering sleep mode and waking only when communication is required, which keeps power consumption low [16]. We focus on two layers of the BLE protocol stack: the Link layer and the GATT layer [20]. From the Link layer, we extract features from broadcast packets and advertisement patterns, while from the GATT layer, we capture the services and characteristics present in the GATT profiles, as illustrated in Table 2.

#### 3.2.1. BLE Broadcast Packet Features

**Figure 2:**
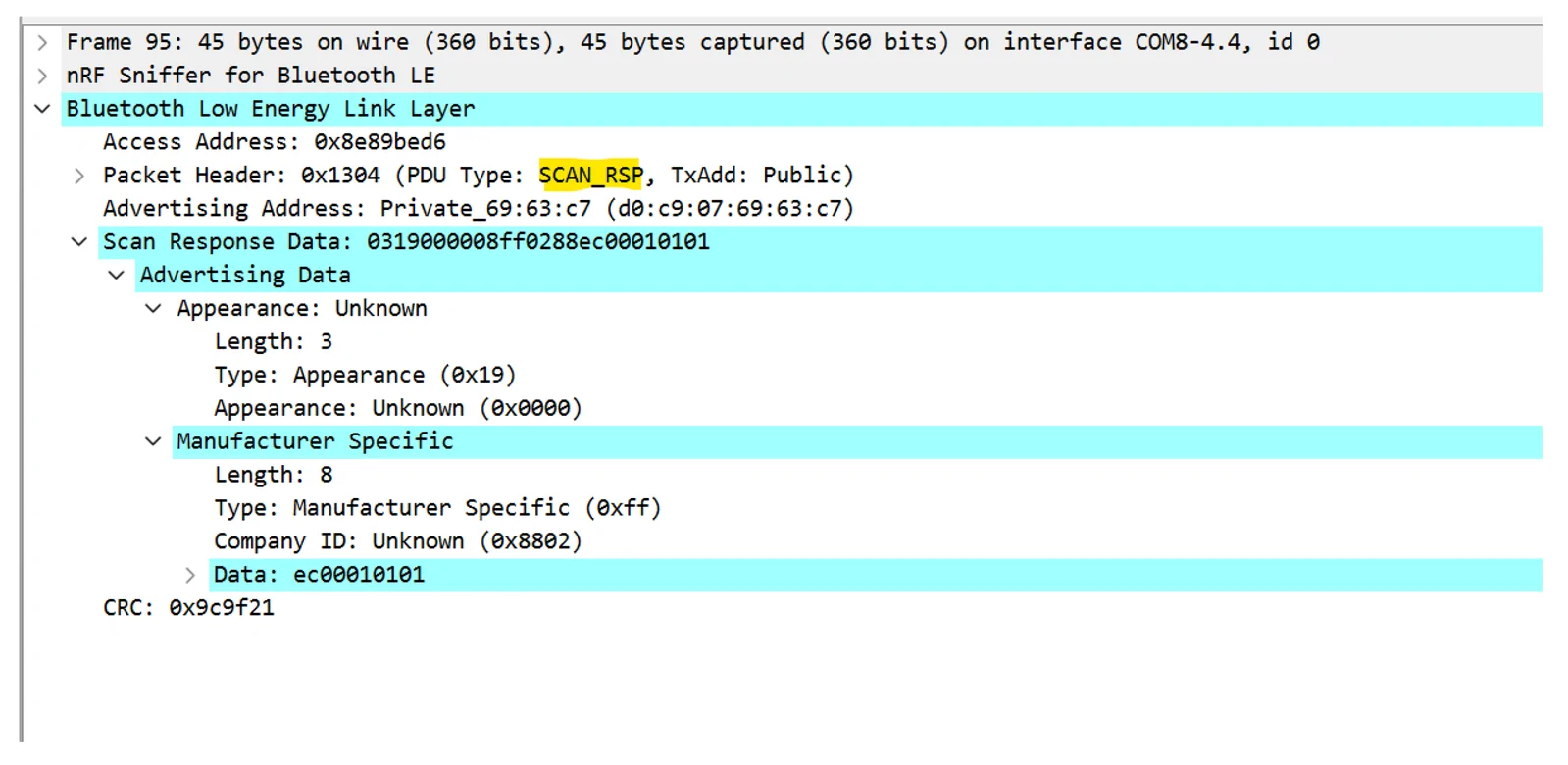
SCAN_RSP Broadcast packet type.

**Figure 3:**
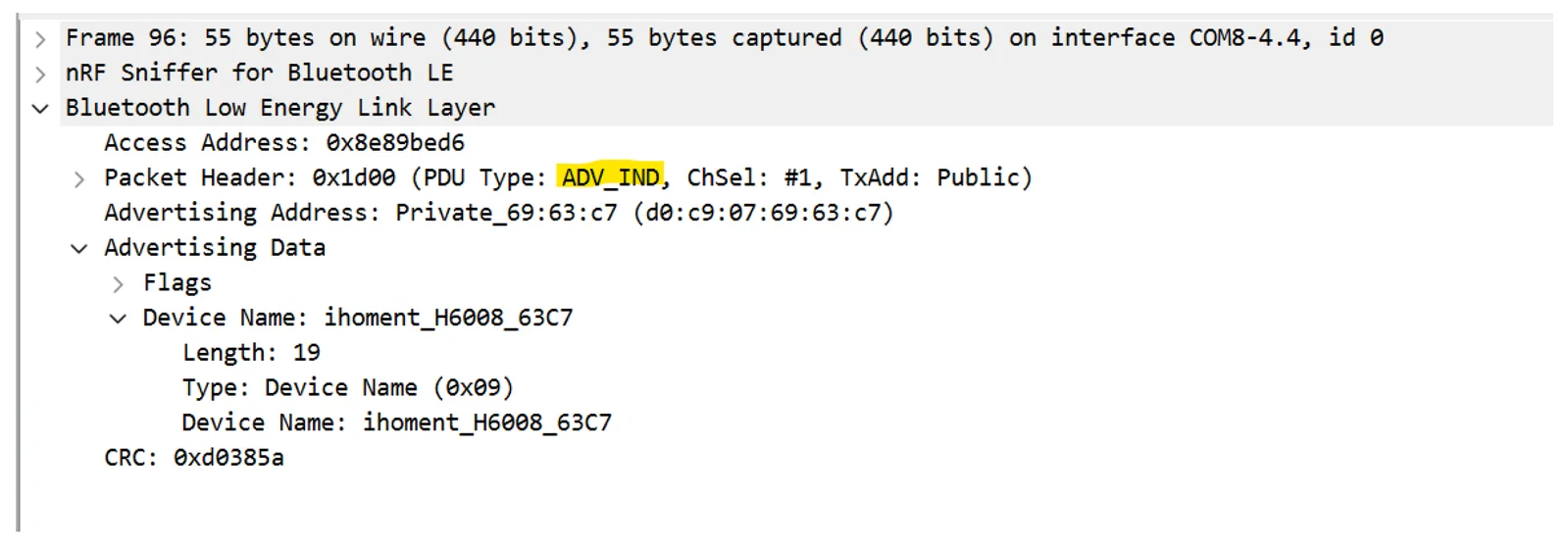
ADV_IND Broadcast packet type.

Broadcasting is the core mechanism for the discovery and connection of BLE devices. Peripheral devices send broadcast packets that central devices detect through scanning, which allows the central devices to discover the broadcasters and initiate connections with them [15]. Our experimental results show that advertising-channel packet types such as ADV_IND, ADV_NONCONN_IND, and SCAN_RSP can effectively support BLE device type-manufacturer identification during the pre-association phase (Figure 2 and Figure 3). Our approach exploits the structure of manufacturer-specific data while selecting features that capture variations across device type-manufacturers.

#### 3.2.2. Advertisement Pattern Features

Peripheral devices typically remain in sleep mode most of the time to conserve energy. However, they must periodically broadcast advertising packets to announce their presence, enabling central devices to discover them. IoT devices emit these packets according to distinct patterns, and once a central device detects a peripheral, it decides whether to establish a connection for data transfer [14]. The advertising event is the fundamental unit for broadcasting advertising packets. In a typical advertising event, one PDU is transmitted on each of the three advertising channels. The event may end after the final advertising PDU is sent, or the advertiser may end it early to free the radio for other tasks. Each IoT device exhibits distinctive advertising patterns that reflect its specific hardware and software attributes.

- **Advertising event interval:** Devices transmit advertising data at varying frequencies depending on their hardware capabilities and functional requirements. The short advertising event interval allows a central device to quickly discover them when data transfer is needed.
- **Advertising channel sequence:** Within each advertising event, advertising PDUs are transmitted over three advertising channels. There are six possible channel order combinations, such as (37, 38, 39) or (37, 39, 38).
- **Advertising delay distribution:** IoT environments typically contain numerous devices that transmit advertising packets at regular intervals. To reduce the likelihood of channel collisions during simultaneous transmissions, advertising events are temporally randomized through the use of advertising delay (adv Delay).

#### 3.2.3. GATT Profile Features

In BLE, the Attribute Protocol (ATT) operates as a stateless client/server protocol organized around attributes, with devices able to assume either role regardless of their BLE role (peripheral or central). The server exposes its data through a GATT profile, a hierarchical structure of attributes that enables information exchange between the client and the server. Within this profile, each service consists of one or more characteristics, and each service and characteristic is identified by a universally unique identifier (UUID) [13]. Besides its UUID, a characteristic includes an attribute handle, properties, and a value. The handle identifies the attribute’s location in the profile, while the value contains its actual data. The properties are metadata that define which ATT operations (such as read or write) are permitted for the attribute, along with any associated security requirements (such as encryption and authentication). Although vendors can define custom services and characteristics, many standard ones are already defined by the Bluetooth SIG [21].

**Figure 4:**
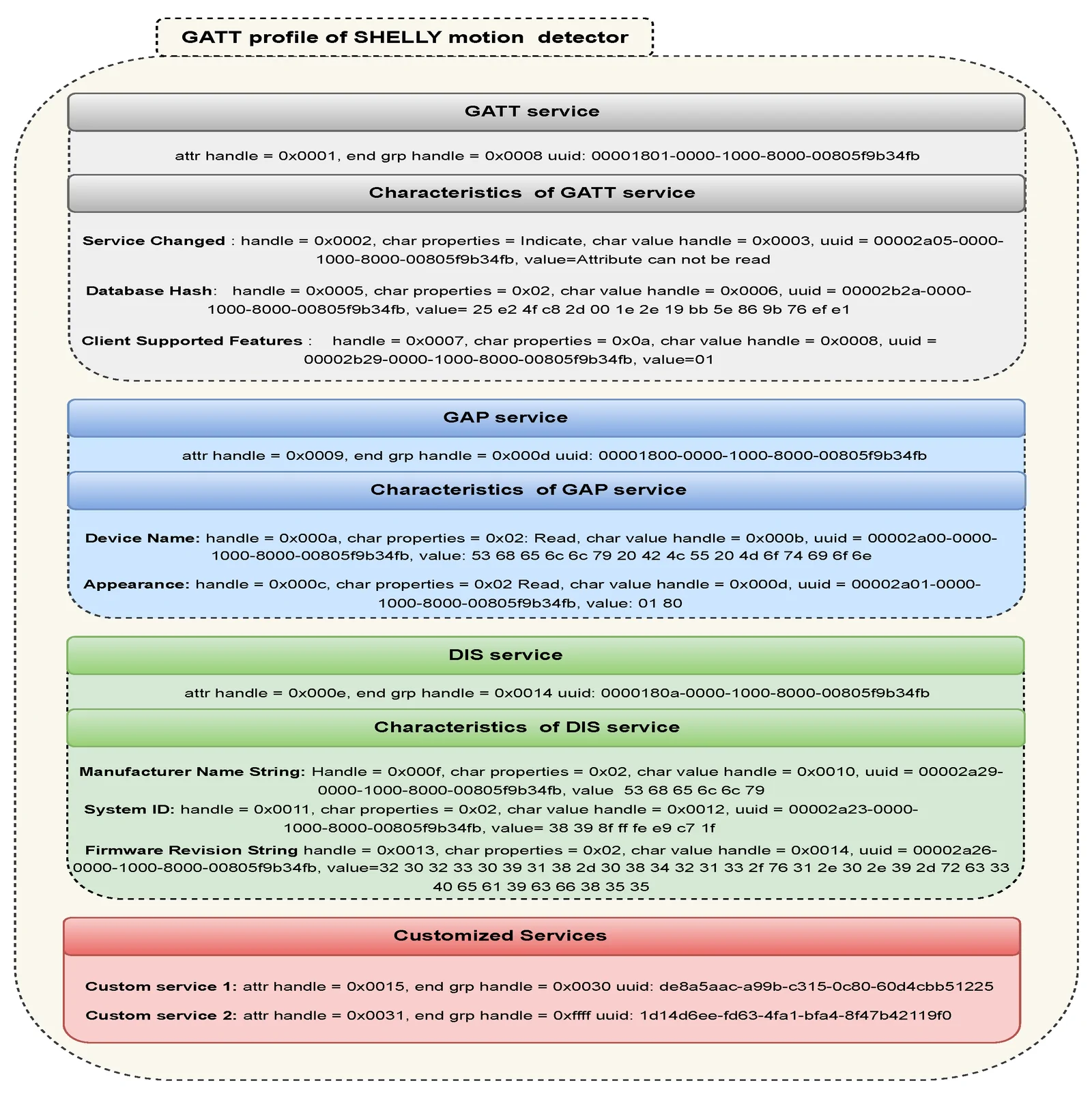
The hierarchy representation of a GATT profile.

In general, a GATT profile is a data structure composed of numerous elements that can vary between devices, which makes it a valuable source for fingerprinting. For instance, a Shelly motion detector we examined contains a profile with three SIG-defined services, as illustrated in Figure 4. For fingerprinting purposes, we consider only custom services and not their individual characteristics, since the service-level structure already provides sufficient distinctiveness to serve as a reliable device identifier.

**Figure 5:**
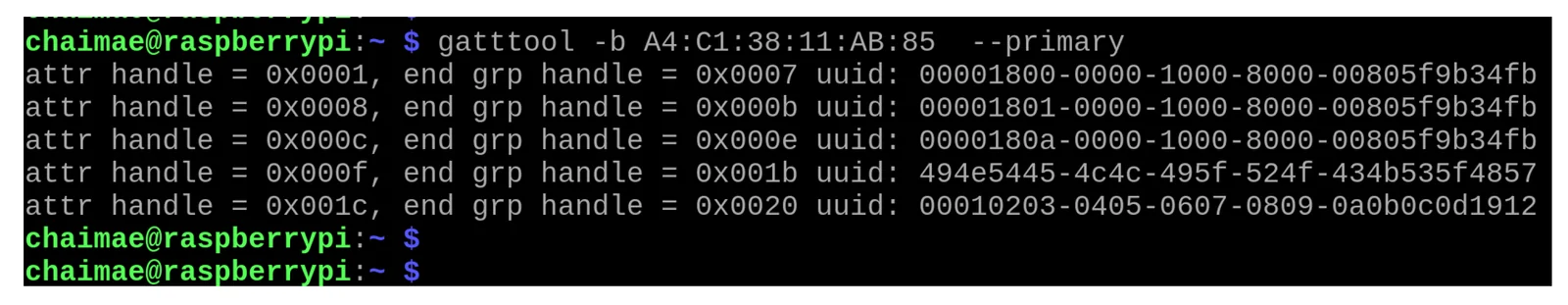
The main services of Govee thermometer using GATTTOOL.

Regarding characteristics, we observed that some devices, such as the Shelly plug and the Govee lightbulb, lack standard services such as the GATT or the Device Information Service (DIS) altogether. Even when these services are present, the number and type of their characteristics vary widely. For example, the DIS on the Shelly device has three characteristics, while the Govee thermometer’s DIS has only the PnP ID. Likewise, for the Generic Attribute service, the Shelly motion detector includes three characteristics, while the Govee lightbulb includes only the Service Changed characteristic. These variations make devices easier to distinguish from one another. Although GATT client/server interactions occur after connection, we found that the list of services and characteristics, along with their associated metadata, can be accessed without pairing or authentication. We extracted these data using Gatttool, as shown in Figure 5.

## 4. Test and Experiment

To evaluate the performance of Nilsimsa locality-sensitive hashing in identifying BLE device type-manufacturers, we employed a two-stage experimental design. The first stage performs an exhaustive combinatorial analysis, evaluating every feature subset to characterize how identification accuracy and hash similarity vary with subset size and composition. The central question motivating this analysis is whether combining link-layer broadcast features (advertising headers, timing patterns) with GATT service information (GAP, DIS, appearance, manufacturer name, System ID) meaningfully improves device-type identification over single-layer approaches, or whether the increased dimensionality confers no discriminative gain. To this end, we evaluate system resilience to incomplete or absent feature sets. In addition, by enumerating and ranking all C(23,k) feature combinations by a composite accuracy–score metric, we determine the smallest feature subset that maintains high identification accuracy and identify the features that contribute most to inter-class separation under the Nilsimsa similarity score.

The second stage examines the top-ranked combinations from the exhaustive search at operationally relevant scale, to verify that performance does not degrade under greater intra-class variability and more diverse device-type distributions. The following subsections describe the experimental methodology and define the evaluation metrics.

### 4.1. Testbed Setup and Dataset Generation

To build a realistic BLE fingerprint dataset and assess the proposed Nilsimsa-based identification system, we assembled an experimental testbed and created a heterogeneous dataset that includes both real-world captures and synthetic data. First, we built a testbed using a Raspberry Pi 3 as an edge gateway and four smart home appliances: a Shelly Plug, a Shelly Motion Detector, a Govee light bulb, and a Govee thermometer. Link-layer features, such as raw broadcast packets and advertising patterns, were collected using a Nordic Semiconductor nRF52840 USB dongle running the nRF Sniffer for Bluetooth LE, with a script automating packet collection and field extraction. In parallel, the GATT service information was probed through Gatttool, which opened a short unauthenticated ATT connection to each device’s GATT server and read the exposed service hierarchy, including the DIS, GAP, GATT, and manufacturer-specific custom services. No pairing, bonding, or authentication was performed: most GATT elements are readable without authentication and before network access is granted, as supported by Celosia et al. [13] and BLENDER solutions [12]. The acquisition therefore occurs before any authenticated association, in contrast to BF-IoT, which derives its ATT/GATT features from session-level traffic collected from the link-establishment stage onward. To simulate the diversity of a high-density IoT environment and evaluate our Nilsimsa-based approach against a broader spectrum of devices, we expanded the collected real-world data into a synthetic dataset comprising 100 type-manufacturer pairs. The synthetic records were generated by modeling the structural and behavioral patterns observed in the real dataset, to simulate realistic IoT network traffic. To evaluate a larger number of classes, we generated 10,000 synthetic records for 100 device type and manufacturer pairs with a Python (version 3.14.6) script, which follows the structure of the testbed records (same fields and formats). Meanwhile, the real testbed records come from 4 type-manufacturer pairs and were duplicated to reach 5000 records. The combined real and synthetic data were partitioned into two sets: a reference dataset of 10,000 records (2500 real captures from the four primary device type-manufacturers and 7500 synthetic records) and a test dataset of 5000 records similar to those in the reference dataset (2500 real and 2500 synthetic). Overall, the exhaustive experiment uses 10 test objects and 10 stored reference hashes, one object per hash, covering 10 device type–manufacturer pairs and including both real and synthetic JSON objects. The large-scale experiment validates the best-ranked subsets on 10,000 reference records and 5000 test records. These datasets cover 104 device type–manufacturer pairs, which correspond to distinct device type-manufacturers: 4 pairs come from the real testbed (Plug–Shelly, Motion Detector–Shelly, Light Bulb–Govee and Thermometer–Govee) and 100 are synthetic. The datasets of both experiments (the 10 reference hashes and 10 test JSON objects of the exhaustive analysis, and the 10,000 reference and 5000 test JSON records of the large-scale evaluation) are publicly available at https://github.com/chaimae-mth/Nilsimsa-dataset (accessed on 2 November 2026).

### 4.2. Exhaustive Combinatorial Feature Analysis

We conducted an exhaustive feature-combination study to evaluate the discriminative power and fingerprint quality achievable under all possible subsets of BLE features within three experimental configurations: (i) Broadcast-only (N = 13 features, $2^{13}\;-\;1\;=\;8191$ combinations), (ii) GATT-only (N = 10 features, $2^{10}\;-\;1\;=\;1023$ combinations), and (iii) a unified Broadcast+GATT feature space (N = 23 features, $2^{23}\;-\;1\;=\;8,388,607$ combinations). The Broadcast features are all presented in Table 2. The selection of the 10 retained GATT variables from the Table 2 was guided by two primary considerations. First, these attributes are the most commonly found across actual devices. This is supported by the empirical study conducted by Celosia et al. [13], which analyzed over 13,000 GATT profiles collected over a five-month period. Their findings indicate the consistent presence of certain service and characteristic elements, including Device_Name, Appearance, Service_Changed, Manufacturer_Name_String, Model_Number_String, Firmware_Revision_String, System_ID, and PnP_ID. The two remaining variables were selected based on our testbed observations: the manufacturer-defined Custom_services are present in all devices of our testbed, and the Peripheral_Preferred_Connection_Parameters attribute contains relevant configuration values that can enhance identification performance when they are present. Retaining frequently present attributes limits the impact of missing data, which is a central concern of our approach. Restricting the GATT side to these 10 variables additionally keeps the exhaustive search space at 8,388,607 subsets, which remains fully practical and ensures comprehensive evaluation.

Each configuration enumerates all non-empty feature subsets of size *k*, for k = 1 to k = N. The 13 broadcast features comprise BLE advertising fields (such as the ADV_IND and SCAN_RSP headers, lengths, MAC address, and CRC) together with timing parameters (channel sequence, event interval, and advertising delay). The 10 GATT features correspond to attributes of the GAP service, the Device DIS, and a custom service. Each feature combination was evaluated by applying it to the test dataset (10 JSON objects), while the stored reference dataset (10 Nilsimsa hashes) remained fixed. Each test Nilsimsa hash was compared with the 10 stored hashes, and the best match was identified by the maximum Nilsimsa similarity score (ranging from −128 to +128, where 128 indicates identical fingerprints). Two metrics were computed per combination, classification accuracy and mean Nilsimsa similarity score, and were combined into a composite score (Equation (1)), which prioritizes correct classification over similarity value.

(1) $$\mathrm{Composite}\;\mathrm{score}\;=\;0.6\;\times \;\frac{\mathrm{accuracy}}{100}\;+\;0.4\;\times \;\frac{\mathrm{mean\_score}}{128}$$

The key aim of a device identification framework is accurate classification. Accordingly, the accuracy metric is given a larger weight (0.6) to highlight the framework’s capacity to match each query device to its reference identity. The parameters are set empirically, giving higher priority to feature combinations that achieve the best identification performance across heterogeneous BLE devices and feature distributions. In this first experiment, we aimed to assess all subsets of BLE features to understand how identification performance changes under incomplete GATT, broadcast, or combined observations. This addresses feature availability rather than dimensionality reduction.

### 4.3. Large-Scale Evaluation of Top-Ranked Feature Combinations

Based on the findings of the exhaustive combinatorial analysis, a large-scale evaluation experiment was conducted to evaluate the scalability and generalization of the top-performing feature subsets. The evaluation was structured as a three-step pipeline applied to a larger dataset comprising 10,000 enrolled BLE device records, which form the stored reference database, and 5000 test records for evaluation. The dataset encompassed 100 device type–manufacturer pairs, including Plug–Shelly, Light Bulb–Govee, Motion Detector–Shelly, Thermometer–Govee, Smartwatch–Apple, Water Bottle–HidrateSpark, and Smart Coffee Maker–Keurig. Note that exhaustiveness, defined as the assessment of all possible feature combinations, is specific to the first experiment. In contrast, the second experiment confines the assessment to the highest-ranked combinations within each subset size K, as determined in the first experiment, to validate their performance at a scale that reflects real-world operational conditions rather than repeating the entire exhaustive search. It evaluates a different hypothesis: whether the obtained rankings remain valid when intra-class variability and device heterogeneity are greater. Selecting the top 100 combinations strikes a balance between comprehensive coverage and computational feasibility: it preserves diverse high-performing candidates across subset sizes while avoiding the computational burden of validating the full combinatorial space on a much larger database.

**Selection of the best combinations**: The best performing feature combinations were selected from the exhaustive combinatorial results based on their composite scores. Combinations were ranked in descending order within each subset size (*k*), with the top 100 combinations retained. For (k = 1), all 23 possible combinations (C(23,1) = 23) were preserved, while for (k = 23), the single full-feature subset was retained. For (k = 22), only 23 combinations were possible (2322 = 23), and all of them were selected. For intermediate sizes k = 2 through k = 21, exactly 100 combinations were kept per subset size, producing a total of 2047 candidate combinations. This selection approach reduced the search space from more than eight million possible combinations to a tractable set of highly discriminative candidates while maintaining representation across the entire range of subset sizes.

**Distribution of the best combinations**: The 5000 test records were distributed across the 2047 top combinations using a deterministic, non-overlapping assignment strategy. Each combination was assigned two or three unique records from the test pool, ensuring that no single record was examined under multiple feature configurations.

**Large-scale Nilsimsa identification and performance evaluation**: Each of the 5000 test records was processed through the Nilsimsa identification algorithm, which proceeds in three sequential stages: JSON cleaning (removal of empty and unreadable values), leaf-value extraction, and Nilsimsa hash computation (256-bit digest). The resulting hash was compared against all 10,000 stored reference hashes using the Nilsimsa similarity score, and the reference record yielding the highest similarity was returned as the identification prediction. The identification was judged correct if the predicted device type-manufacturer matched the ground-truth device type-manufacturer.

The identification efficacy was characterized by computing macro-averaged accuracy, precision, recall, and F1-score across the 100 device types-manufacturers for each combination size k ∈ K, where K = {1, 2, …, 23}, using the Step 3 identification outputs. Macro-averaging was deliberately chosen over weighted aggregation so that every device type-manufacturer contributes equally to the reported scores, regardless of how frequently it appears in the test set, which is a critical property given the inherent class-size heterogeneity of real-world BLE deployments. The resulting metrics were examined through three complementary visualizations: overall performance curves tracking how each metric evolves as *k* increases; grouped per-device-type bar charts disaggregating precision, recall, and F1-score within three combination-size partitions (k = 1–8, k = 9–17, and k = 18–23); and line plots tracing the metric trajectory of each device type-manufacturer across those partitions. Collectively, these visualizations facilitate a granular assessment of identification robustness, revealing how feature quantity and modality composition jointly influence both coarse-grained accuracy and fine-grained per-class discrimination in large-scale BLE fingerprinting scenarios.

The identification task addressed in this work is a multiclass problem: each of the 5000 test records is compared against a reference database of 10,000 stored Nilsimsa hashes representing 100 distinct device type/manufacturer pairs, and the single highest-similarity match is returned as the prediction. To obtain interpretable per-type performance metrics, we decompose this multiclass task into a collection of one-versus-rest binary classification problems. For each device type-manufacturer *d*, true positives TP_d count correct identifications of type *d*; false positives FP_d count instances where another type is misclassified as *d*; false negatives FN_d count instances where *d* is misclassified as another type; and true negatives TN_d encompass all correct rejections of non-*d* types.

Precision (Equation (2)), recall (Equation (3)), and F1-score (Equation (4)) are then derived from these per-type counts and subsequently macro-averaged across all 100 device types-manufacturers. The averaging is termed “macro” because we compute the evaluation metric independently for each class before taking the arithmetic mean of the resulting values, thereby assigning equal importance to every device type-manufacturers regardless of its frequency in the dataset. By weighting all classes equally, macro-averaging prevents frequently occurring device type-manufacturers from dominating the overall evaluation, providing an unbiased assessment of the proposed identification framework across heterogeneous BLE device type-manufacturers.

(2) $$P_{d}=\frac{{\mathrm{TP}}_{d}}{{\mathrm{TP}}_{d}+{\mathrm{FP}}_{d}}$$

(3) $$R_{d}=\frac{{\mathrm{TP}}_{d}}{{\mathrm{TP}}_{d}+{\mathrm{FN}}_{d}}$$

(4) $$F1_{d}=\frac{2P_{d}R_{d}}{P_{d}+R_{d}}$$

Macro-averaged metrics are computed as unweighted means:

(5) $$\begin{array}{c} \mathrm{Mean-P}=\frac{1}{\left|D\right|}\sum_{d\in D}P_{d},\;\mathrm{Mean-R}=\frac{1}{\left|D\right|}\sum_{d\in D}R_{d},\\ \mathrm{Mean-F}1=\frac{1}{\left|D\right|}\sum_{d\in D}F1_{d}, \end{array}$$

where D is a set of device type-manufacturers.

## 5. Results and Discussion

The two evaluation experiments presented in the previous section were designed to examine the behavior of the proposed Nilsimsa-based device identification framework as a function of feature-subset size and composition. First, how do identification accuracy and match confidence, defined respectively as classification correctness and mean Nilsimsa similarity score, scale with the number of features *k*? And does this scaling apply uniformly across feature domains? Second, how many features are required for accurate and reliable identification? This question is particularly relevant for reducing the amount of data that must be captured and processed on resource-constrained gateways, as well as for strengthening the system’s resilience in scenarios where some features are missing. Furthermore, we seek to identify the specific feature subsets that provide the best discriminative performance and to compare the GATT, Broadcast, and Combined feature spaces to investigate whether information from the two protocol domains is complementary. The exhaustive analysis establishes the theoretical best-case convergence behavior of the proposed algorithm across the GATT, Broadcast, and Combined feature domains, while the large-scale study evaluates whether these convergence behaviors remain consistent when the framework is applied to a substantially larger and more heterogeneous dataset. The latter experiment therefore builds on the exhaustive analysis by evaluating the algorithm under more realistic deployment conditions and determining whether the feature-efficiency and confidence scaling observed in the controlled setting generalize to large-scale, heterogeneous device populations.

**Figure 6:**
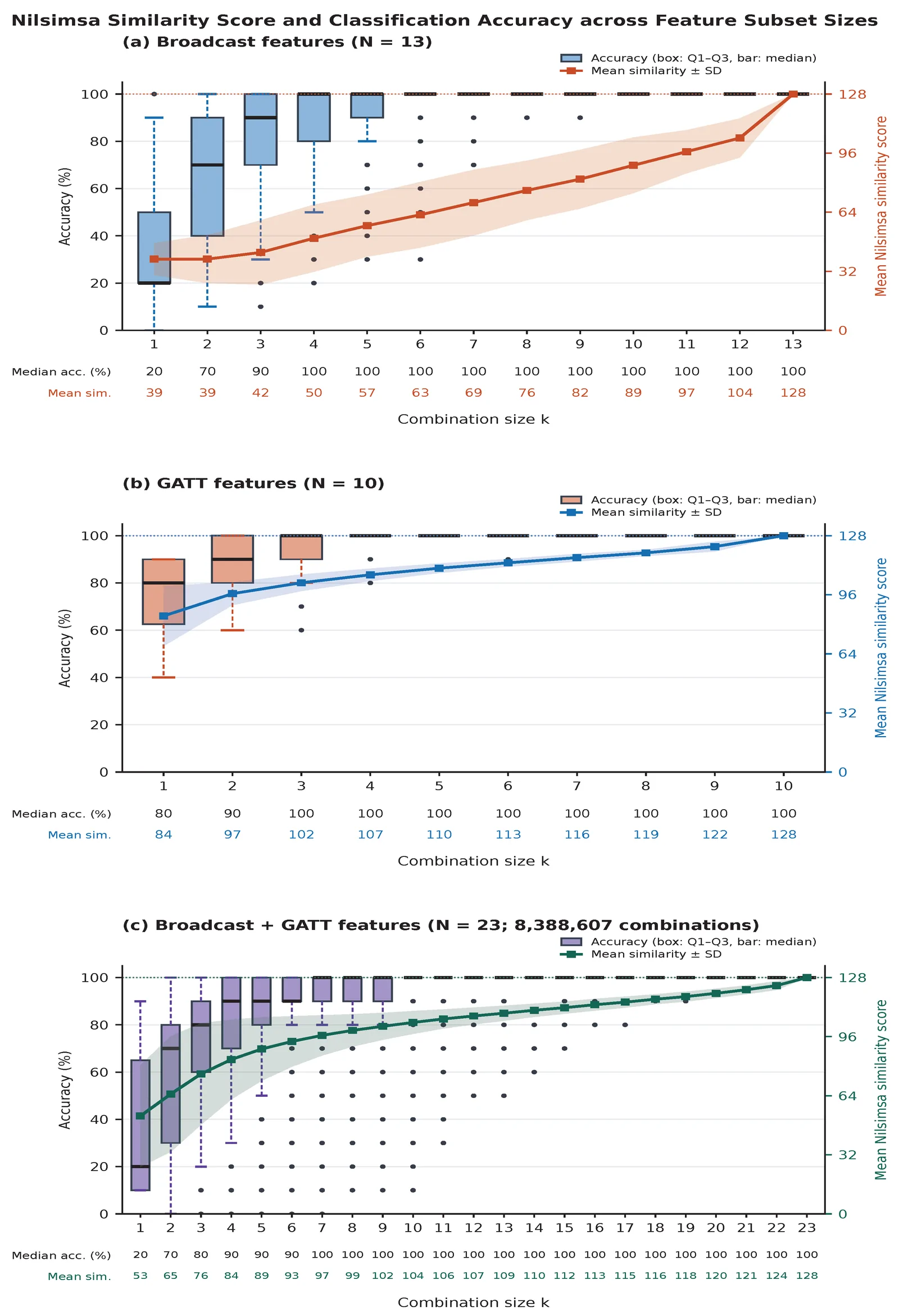
Accuracy distribution and mean Nilsimsa similarity score per combination size *k*. (**a**) Broadcast features (N = 13); (**b**) GATT features (N = 10); (**c**) Broadcast + GATT features (N = 23; 8,388,607 combinations). Boxes show the accuracy quartiles (Q1–Q3) with the median as a bar; the line shows the mean similarity score, and the shaded area shows ±1 standard deviation.

**Figure 7:**
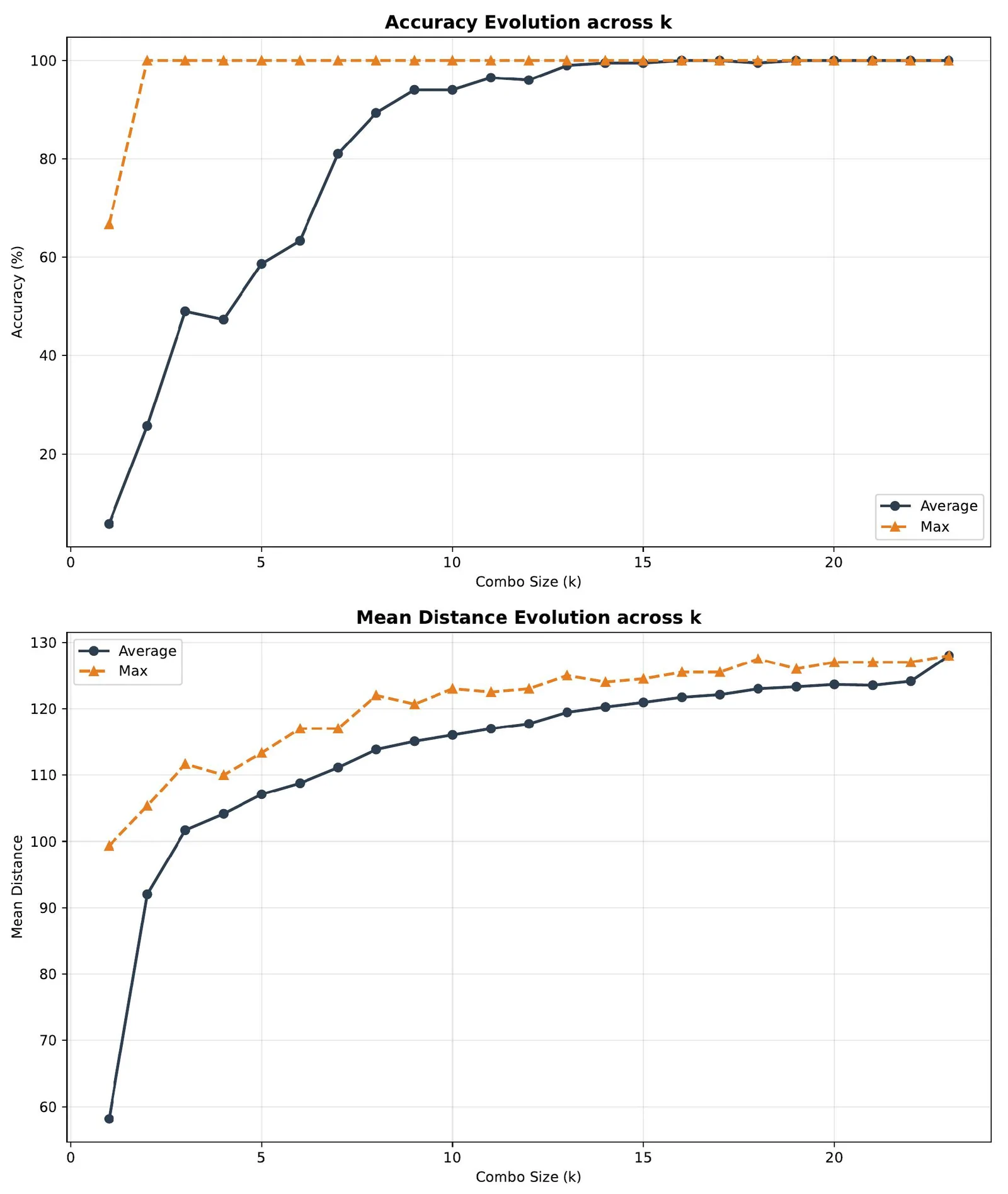
Evolution of identification performance metrics across combination sizes (k).

On the broadcast side, Figure 6 shows that individual advertising fields are heterogeneous and noisy at small *k*, exhibiting a wide interquartile range with only 20% median accuracy at k = 1; however, performance improves rapidly, reaching 100% median accuracy at k = 4 and a perfect Nilsimsa similarity score of 128 at k = 13, indicating that a key subset of roughly four broadcast attributes is sufficient for precise device type-manufacturer fingerprinting. The GATT results show rapid convergence, with a single characteristic reaching a median identification accuracy of 80% and a mean Nilsimsa similarity score of approximately 65. Increasing the subset size to k = 3 raises the median accuracy to 100%, accompanied by a Nilsimsa similarity score of 97. This strong performance indicates that manufacturer- and model-specific characteristics, such as Device_Name, System_ID, and PnP_ID, contain highly discriminative information that can distinguish device type-manufacturers using a small number of features. In contrast, broadcast parameters such as advertisement delay, channel sequence, and CRC mainly capture temporal or protocol-level differences that are largely independent of device identity and hence have little discriminative value for device classification. For the Combined feature space, convergence occurs at a higher subset size, with 100% median accuracy achieved only at k = 9, while the mean Nilsimsa similarity score increases progressively from 53 at k = 1 to 128 at k = 23. As shown in Figure 7, the large-scale test follows the same pattern as the exhaustive study: accuracy and Nilsimsa similarity score improve as more features are added. However, it requires larger feature sets to reach the same results. Accuracy reaches about 94% at k = 9 (compared with 100% in the first experiment) and attains 100% at around k = 14–15. The mean Nilsimsa similarity score also grows, from about 58 at k = 1 to 128 at k = 23. The lower accuracy observed in the large-scale study is attributable not to limitations of the evaluation metric but to the greater complexity and heterogeneity of the 5000-record dataset, which introduces more challenging cases than those represented in the smaller experimental dataset. Moreover, the larger sample provides more stable and reliable performance estimates by reducing the influence of sampling variability. Thus, the approximately 94% accuracy observed in the large-scale experiment provides a more robust indication of the framework’s general performance, whereas the 100% accuracy achieved in the smaller exhaustive study may partly reflect the comparatively limited and less challenging evaluation population. It should be noted that the three feature groups are evaluated with different reference setups. For the broadcast-only and GATT-only subsets, the reference hash is built from the same features as the query. In this case the Nilsimsa similarity can reach 128 (for example, at k = 13 for broadcast), because the query and the stored record are identical when all features of the group are used. For the combined subsets, the reference hash is built from all 23 features, and the query uses only the selected subset. The similarity therefore reaches 128 only at k = 23.

**Figure 8:**
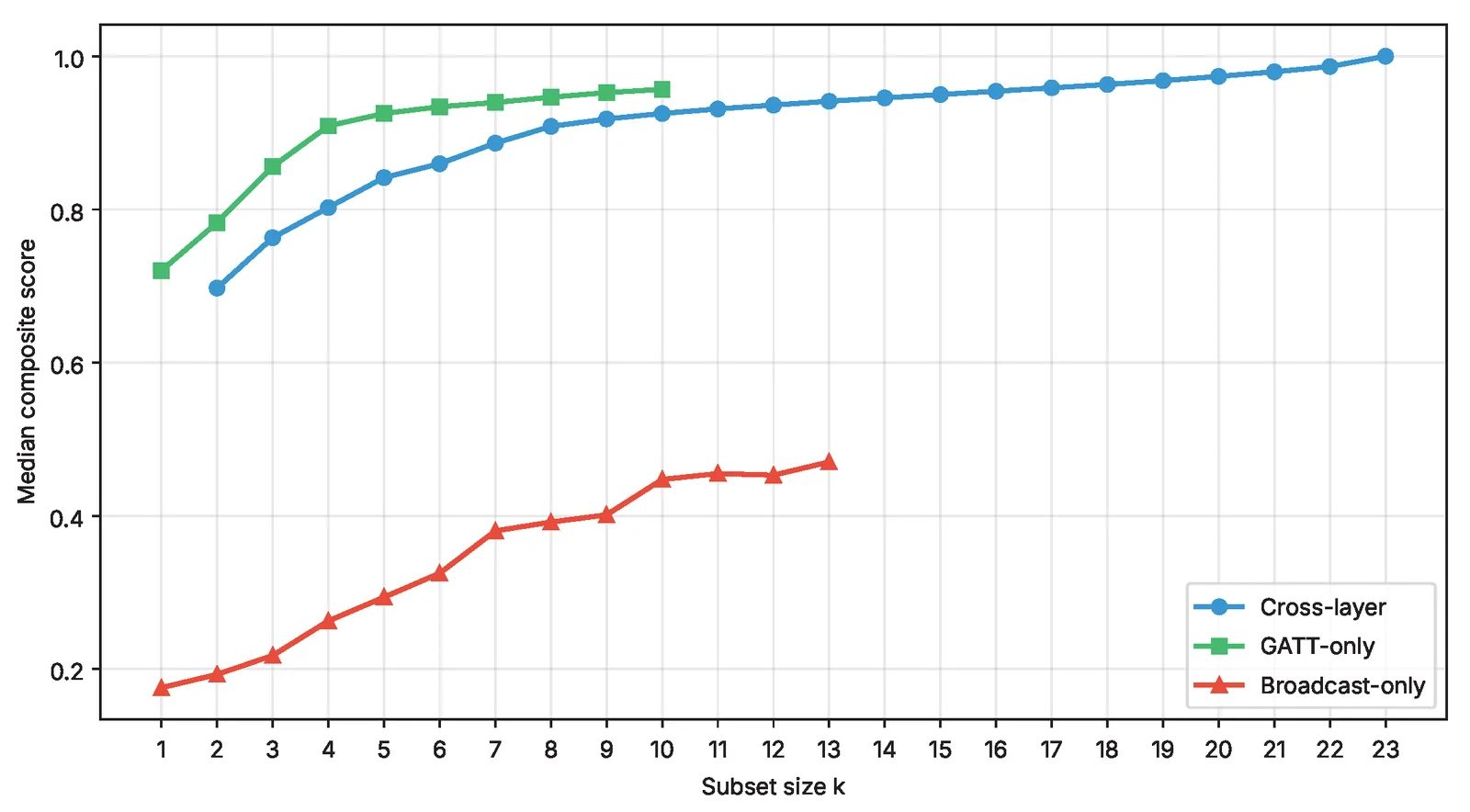
Composite score per k size within the combined features space C(1,23).

Figure 8 compares the three types of subsets at the same size k, using the median composite score over all 8,388,607 subsets of the exhaustive experiment (combined features space). A curve stops where no subset of that type exists (GATT-only after k = 10, broadcast-only after k = 13). GATT-only subsets have the highest median at every size where they exist (k = 1 to 10), from 0.72 to 0.96. Broadcast-only subsets are substantially lower, rising from 0.18 at k = 1 to only 0.47 at k = 13, which is below a typical single GATT attribute. Cross-layer subsets are close to GATT-only subsets but below them (gap of 0.09 at k = 2, 0.11 at k = 4 and 0.03 at k = 10). From k = 14, only cross-layer subsets exist, so no comparison is possible. A possible reason for the weight of GATT attributes is that each one adds several fields to the fingerprint (handles, UUID and value), whereas a broadcast attribute is most of the time a single short value. Although GATT features give the highest accuracy at equal subset size, this does not make the broadcast layer unnecessary. In our testbed, the attributes available for a device are not always the same: some devices expose few GATT attributes, and there is no fixed structure across device types and manufacturers. Broadcast attributes are read without a connection, so they remain available when a device exposes only part of its GATT profile. In such cases, a method that uses both layers can still produce a fingerprint, while a GATT-only method cannot.

**Figure 9:**
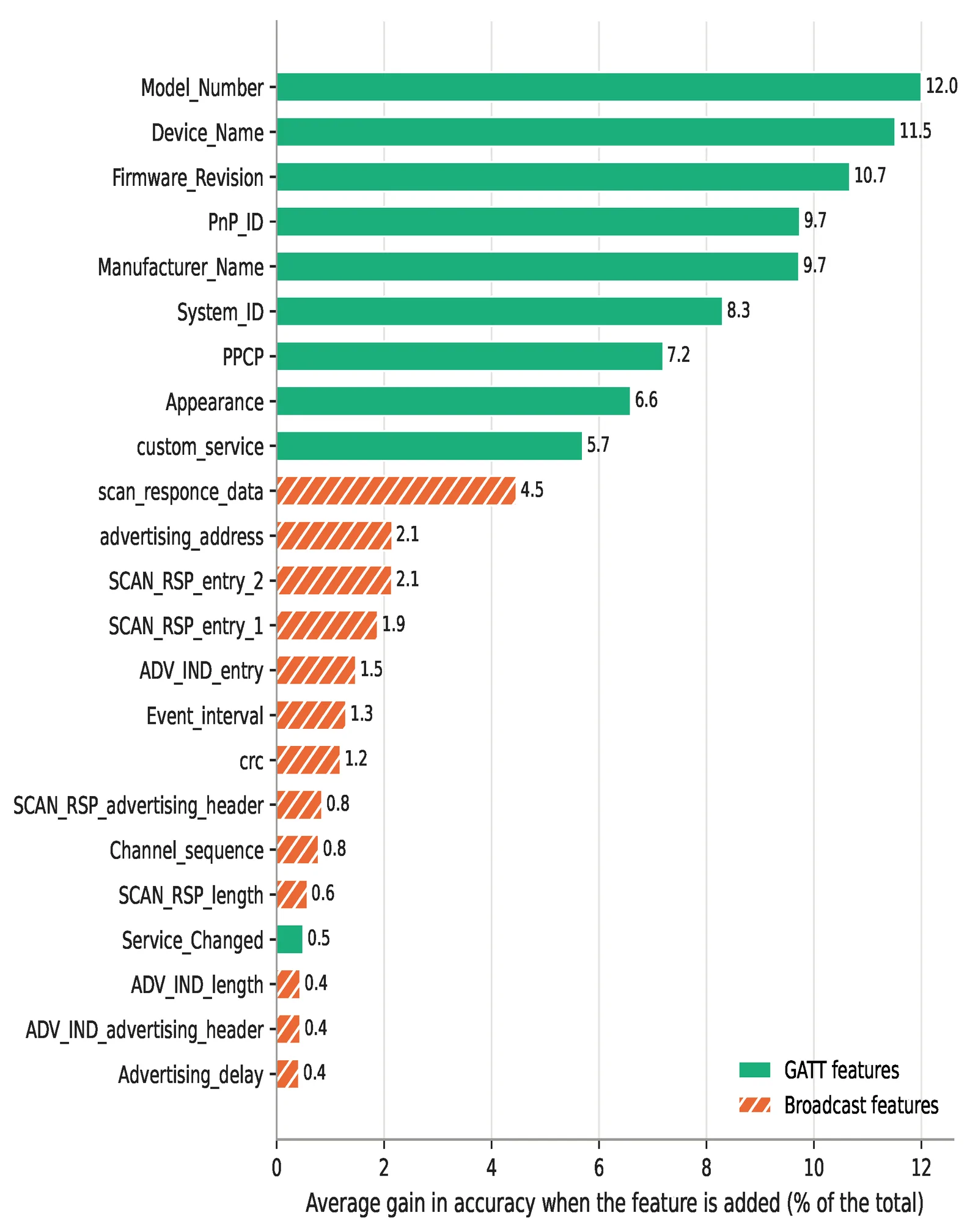
Feature importance within combined feature subsets.

To measure the importance of different features, we use two quantities computed on all 8,388,607 subsets of the exhaustive experiment. The first is the accuracy of the feature alone (a subset of size 1). The second is the mean change in accuracy when the feature is added to a subset of size K that does not contain it. For each size K, we compare every such subset with the same subset plus the feature, so the comparison is paired and uses no sampling. We report this mean change for small subsets (K = 1–8), medium subsets (K = 9–17) and large subsets (K = 18–22), and as an average over all sizes. In this average, every size from 1 to 23 has the same weight, and size 1 is the feature alone. Figure 9 demonstrates that GATT characteristics dominate individual contributions, with the Model_Number_String, Device_Name, and Firmware_Revision attributes contributing 10.7%, 9.7%, and 9.7% of the total gain, respectively, followed by PnP_ID (8.3%), Manufacturer_Name (7.2%), System_ID (6.6%), PPCP (5.7%), and Appearance (4.5%). The 13 broadcast features contribute smaller individual gains but jointly account for approximately one third of the total, confirming that neither layer alone reaches the performance of the full cross-layer set. It should be noted that a GATT attribute also contributes several fields (handles, UUID and value) to the fingerprint, whereas a broadcast attribute is one short value, and this explains part of the difference between the layers.

**Figure 10:**
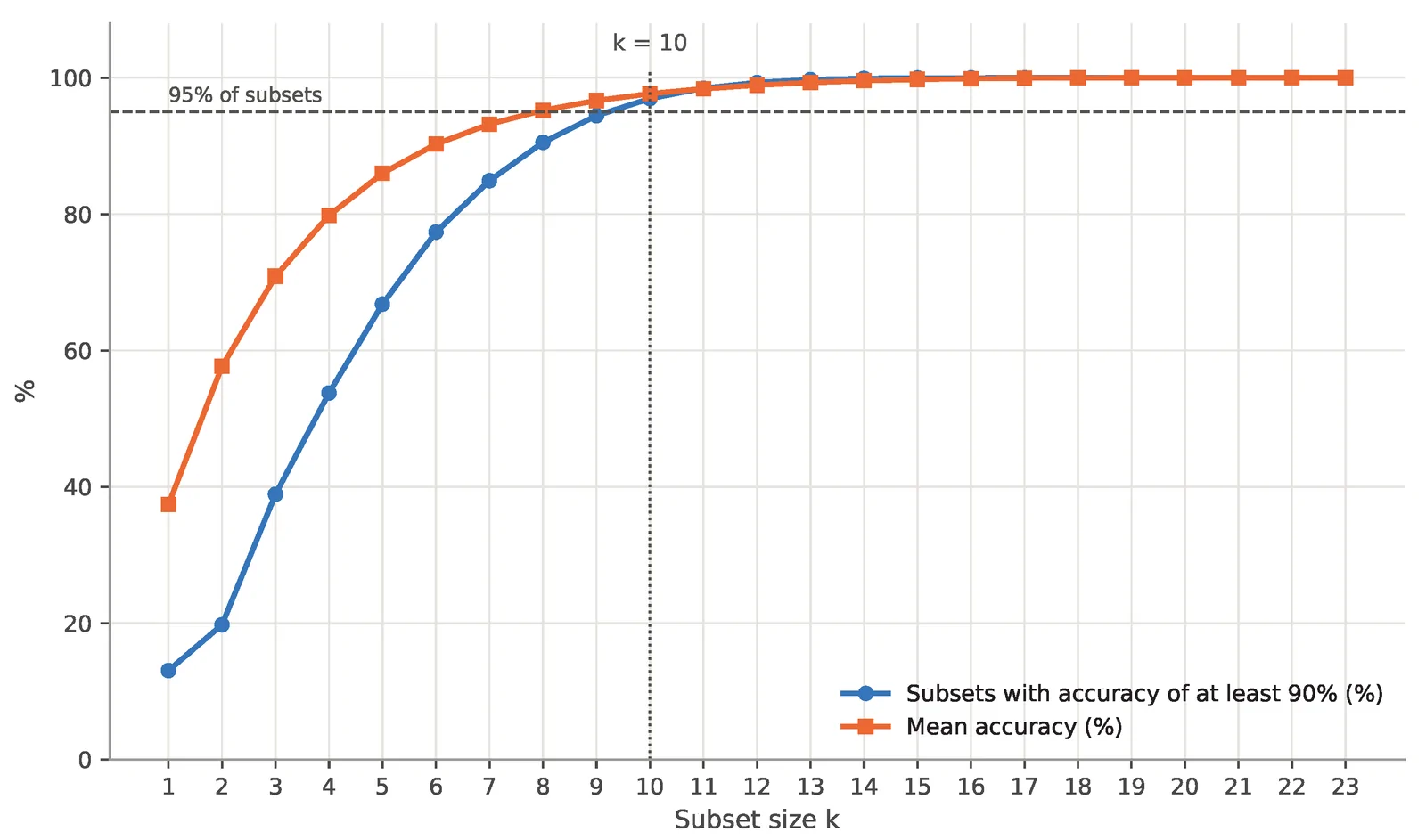
Contribution of each feature to identification accuracy.

To identify the minimum sufficient feature count, we fixed the following criterion before examining the results: a subset size *k* is sufficient when at least 95% of all subsets of that size reach an identification accuracy of at least 90%. Figure 10 shows that the minimum is k = 10: 97.0% of the 1,144,066 subsets of size 10 reach 90% accuracy, against 94.4% at k = 9 and 90.5% at k = 8, and the mean accuracy at k = 10 is 97.7%. Cross-layer subsets need k = 10, and more than 99.9% of the subsets of size 10 are cross-layer, so the k = 10 result primarily reflects the behavior of cross-layer subsets. Cross-layer subsets therefore need more features than GATT-only subsets, because many added broadcast features bring important accuracy. Their value is that a fingerprint can still be built when GATT attributes are missing: in our testbed, some devices lack the DIS service, some expose no GATT service, and some do not broadcast a device name or company identifier.

**Figure 11:**
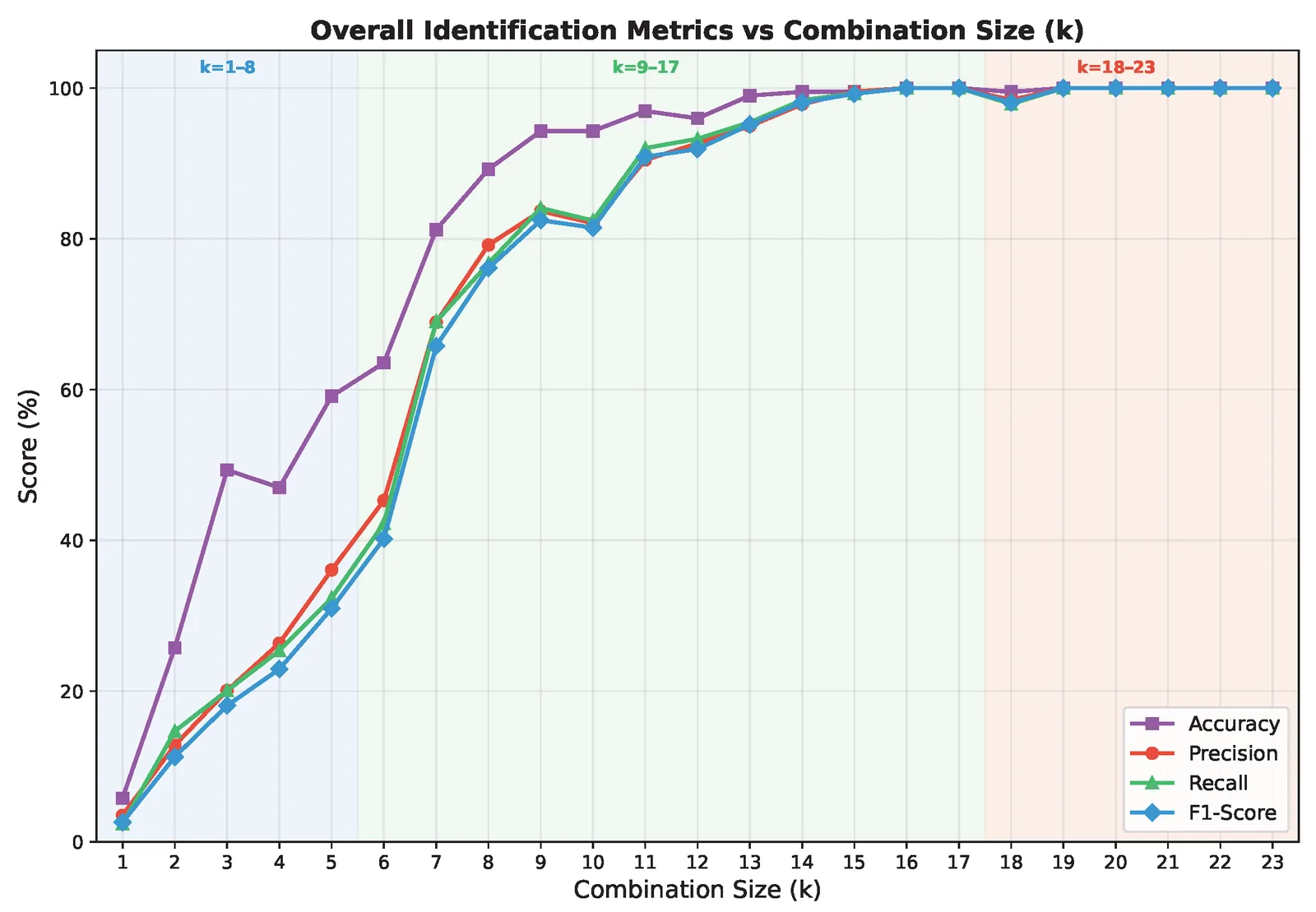
Variation of accuracy, precision, recall, and F1-score with the number of combined features k.

**Figure 12:**
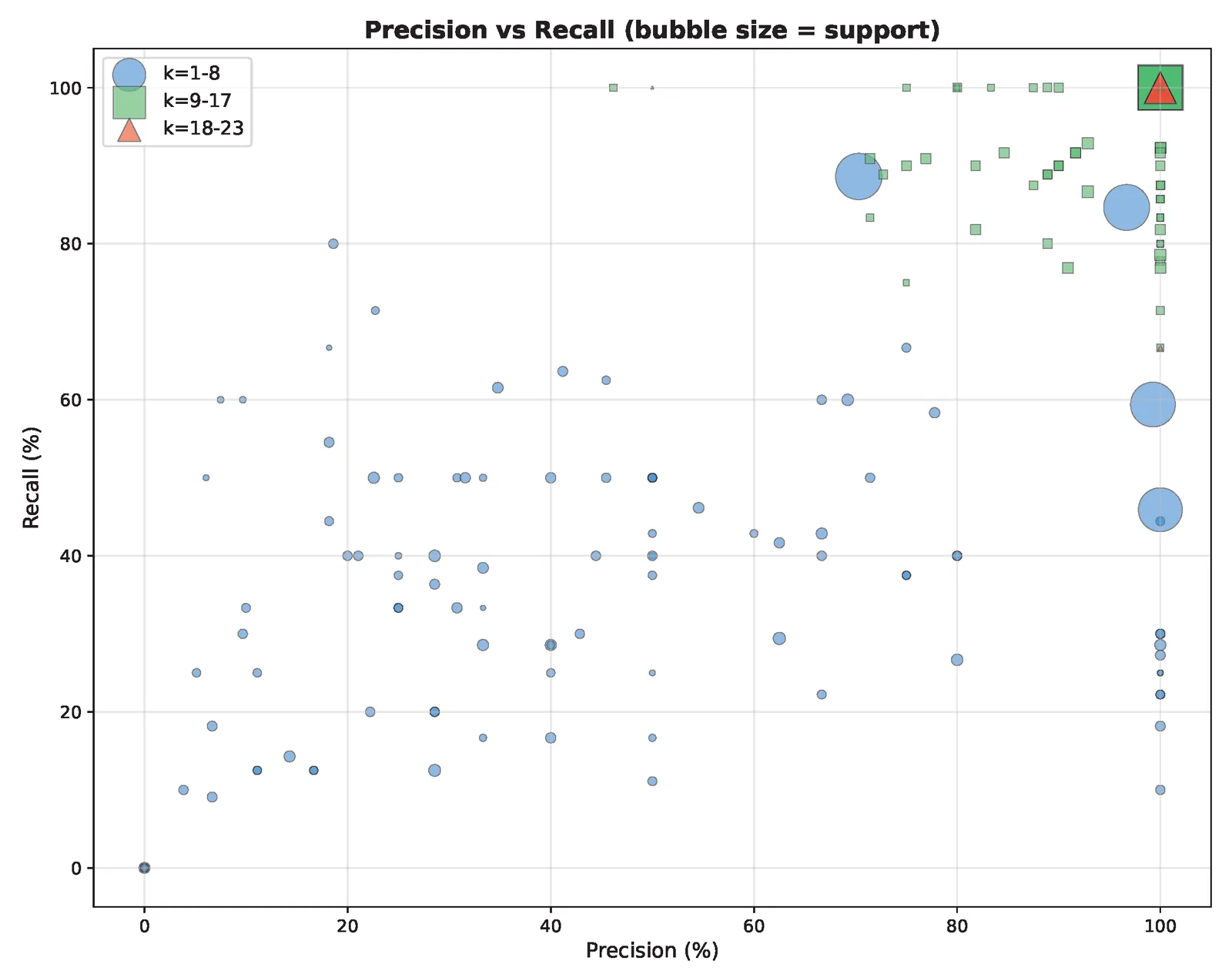
Per-class precision–recall distribution across feature combination sizes.

Figure 11 examines how BLE device type-manufacturer identification performance evolves as the number of features *k* increases in a realistic high-volume deployment scenario. Beyond overall performance, it also assesses whether expanding the feature set introduces systematic biases toward over-prediction, where frequent false positives (FP) degrade precision, or toward under-prediction, where frequent false negatives (FN) reduce recall, for any given device type-manufacturer. To provide a comprehensive assessment, four complementary metrics are considered: overall identification correctness (accuracy) and cross-type performance measured using macro-averaged precision, recall, and F1-score. By weighting all device type-manufacturers equally regardless of sample size, macro-averaging offers a more balanced evaluation of classification performance. This consistency signifies that the improvement is balanced rather than driven by one error type at the expense of the other: incorrect assignments (affecting precision) and missed identifications (affecting recall) decrease together. From a network-security perspective, this convergence is encouraging because it suggests that richer cross-layer fingerprints can improve identification while maintaining a relatively balanced error profile across device type-manufacturers, which is also useful for supporting isolation and access control within IoT networks. Figure 12 complements this aggregate view by examining precision–recall behavior per device type-manufacturer. It plots precision against recall, using bubble size to denote support, and groups the data into three intervals: k = 1–8, k = 9–17, and k = 18–23. The distribution of data points across the three *k* intervals reveals a tendency for most device type-manufacturers to shift toward the upper-right region as *k* increases, indicating simultaneous improvement in both precision and recall rather than a trade-off between them. Taken together, the two figures indicate that incorporating additional features initially improves aggregate correctness and then strengthens confidence in class-level separation, while the stable performance already visible from about k = 9 suggests that the method remains reliable even when some features are absent owing to the diverse, manufacturer-specific attribute configurations of real-world BLE devices.

Prior BLE fingerprinting studies have predominantly relied on a single protocol layer, using broadcast content, GATT profiles, or UUIDs as independent sources of identification features, rather than systematically exploring the joint feature space, and none have combined flow-level and packet-level evidence within a single fingerprint. Although BF-IoT [14] combined link-layer and advertised GATT service information, its cross-layer fusion was implicit within a Random Forest classifier, required pairing and authentication to the device in question, and did not isolate, rank, or exhaustively evaluate the specific feature combinations responsible for performance gains. To the best of our knowledge, our work is therefore the first to exhaustively explore the joint feature space formed by GATT characteristics, broadcast packet fields, and their fusion, while also determining the minimum subset required for reliable identification. The results illustrate that cross-layer features are important to cope with missing features. Moreover, adding features improves not only accuracy but also the confidence margin of each match: in every configuration, accuracy saturates well before mean Nilsimsa similarity score does, so additional features beyond the accuracy-optimal subset continue to push the mean Nilsimsa similarity score toward the perfect-match baseline of 128, indicating stronger separation from incorrect references and enhanced robustness against missing or corrupted features.

Our proposed Nilsimsa method, a technique for identifying similarities among nearest neighbors, offers notable advantages compared to previous ML/DL methodologies in the context of BLE device fingerprinting. Unlike prior approaches that depend on a trained Random Forest model, a fixed 200-dimensional normalized vector and an MLP classifier, or protocol-specific GATT, UUID, GAP, or pairing and bonding to the device in question, our identification system can accommodate unseen device type-manufacturers through simple feature extraction, hash generation, and reference-database registration. This can be achieved without the necessity of retraining a classifier, optimizing new model parameters, or adjusting the core identification algorithm. Furthermore, Nilsimsa’s distinctive strength lies in its approach to data-agnostic aggregation compared to the previously proposed algorithms. By computing locality-sensitive hashes directly from raw feature concatenation, the algorithm retains the granular discriminative patterns that distinguish device implementations across vendors. Its capability to aggregate both structured and unstructured data types from GATT profiles and broadcast packet features without extensive feature extraction or normalization preserves the rich semantic information embedded within these diverse data sources, thereby retaining semantic details such as manufacturer-specific service hierarchies and custom characteristics that may otherwise be obscured during extensive preprocessing. For instance, GATT profiles expose manufacturer-specific service hierarchies and custom characteristics that contain implicit behavioral signatures; preprocessing normalization would necessarily abstract or eliminate these fine-grained distinguishing properties. The practical benefit of preserving these discriminative patterns is reflected in Figure 6, where the GATT accuracy and Nilsimsa similarity score curves converge more rapidly than the broadcast curves, indicating that Nilsimsa effectively exploits the rich identification information contained in GATT attributes. Furthermore, this design enables seamless adaptability to novel firmware updates, emerging device features, protocol extensions, or completely new protocols since only the feature-extraction module needs to be updated rather than complete system redesign, making it more practical and scalable than previous solutions for dynamic and evolving IoT ecosystems. In addition to the advantages offered by Nilsimsa, which suit the IoT BLE environments targeted by our approach, the choice of Nilsimsa is supported by the comparison made by research [22]. They compared two locality-sensitive hashes, Nilsimsa and TLSH, for IoT device identification on three datasets (setup, idle and active states), and they retained Nilsimsa because it gave better identification results.

Across all three experimental settings, the plots illustrated in Figure 6 therefore indicate not only whether a device type-manufacturer is matched, but also how closely the observed fingerprint aligns with the stored reference hash, which makes the decision boundary more informative than a simple label comparison. In practical terms, the Nilsimsa similarity score distribution can serve as an alarm baseline: any identification event with a Nilsimsa similarity score significantly below the expected median can be flagged as a potential impersonation attempt, enabling a two-tiered detection pipeline: (1) type identification, (2) confidence gating. In addition, combining features from the link layer and the GATT layer creates a meaningful security margin, as a potential attacker would be required to simultaneously spoof or suppress identification artifacts across both the link-layer broadcast and GATT service layers. Consequently, cross-layer spoofing becomes substantially more complex than attacking either layer in isolation.

The experimental results presented in Figure 6 and Figure 7 illustrate that a value of k = 9 is where the overall feature space achieves near-saturated median accuracy. On the other hand, the evaluation of Precision, Recall, and F1-scores depicted in Figure 11 indicates that k = 11 offers a more reliable operational threshold for maintaining balanced performance across different classes. While at k = 9, the aggregated metrics range between 82% and 84%, they show improvement to approximately 91% to 92% at k = 11, progressively approaching complete convergence at around k = 15–16. This per-class pattern is consistent with the balanced behavior observed in the macro-averaged results of Figure 11, indicating that a sufficiently rich feature combination, beginning around k = 9, is needed to achieve reliable performance across device type-manufacturers. While previous BLE solutions emphasize choosing a particular set of specific features, our work tackles the missing-features issue that is inherent to BLE devices. As classical solutions based on the “top N” attributes are still static and rigid, in real-world environments, BLE devices do not conform to a fixed set of attributes as we observed in our experiments. Our findings reveal that high identification accuracy is not bound to a static combination of attributes; rather, a subset of 9 features composed of GATT service structures and Broadcast advertising packets, is sufficient to achieve reliable device type-manufacturer identification (with an accuracy of ≥94%) and strong Nilsimsa similarity scores.

## 6. Conclusions and Future Work

Reliable identification of BLE device type-manufacturers serves as a fundamental security measure in the context of secure IoT network administration. This functionality supports specific access controls and vulnerability evaluations tailored to IoT devices, as well as the establishment of behavioral norms specific to each device type-manufacturer to identify anomalies. Such capabilities are crucial within environments with limited resources, where traditional cryptographic or computationally intensive security protocols may pose challenges for implementation. In response to the aforementioned challenge, we have introduced a Nilsimsa-based similarity identification system tailored for BLE device types and manufacturers, combining broadcast advertising features, advertisement timing patterns, and advertised GATT service information. Two experiments addressed a question left open by prior BLE fingerprinting work: whether cross-layer feature fusion meaningfully improves identification over single-layer approaches, and what minimal feature subset sustains high accuracy under the incomplete, heterogeneous feature exposure typical of real-world settings. Through these experiments, we exhaustively examined feature combinations across 104 device-type/manufacturer pairs and validated the best-performing combinations under increased device and feature variability. Key findings highlight that cross-layer features are important to cope with missing features. The minimum subset size was found at k = 10 with a subset-coverage criterion: at least 95% of all subsets of 10 features reach an accuracy of 90% or more. In practice, a device may not expose all 23 features in an ideal scenario. Our exhaustive results show that this case is handled: a subset of 10 features is enough for near-saturated accuracy, and 3 to 5 features from each layer can give high accuracy. When some features are missing, the remaining broadcast and GATT features still identify the device type-manufacturer with high accuracy.

Our approach enables gradual device enrollment by using reference-hash registration, without needing to retrain the core identification algorithm. While currently tailored for BLE, its adaptable feature-extraction layer can be adjusted for new BLE attributes and protocol structures, without overlooking the similarity score-based confidence metric that can support a dual-stage pipeline for type identification, coupled with confidence gating for anomaly detection/spoofing workflows by selecting an optimal threshold. Spoofing and impersonation are known attacks on BLE. An attacker can copy the MAC address of a device, and the GATT services and characteristics can be cloned so that a malicious device pretends to be the original peripheral [1]. Fingerprints that do not rely on identifiers such as IP and MAC addresses help here, because these identifiers are easy to spoof [10]. Identifying the device type and manufacturer from the content of the broadcast and GATT layers gives the expected identity of a device. A device that claims one identity but gives a fingerprint of another type, or a low similarity score, can then be flagged. Cross-layer fingerprints also make the attack harder, because the attacker must copy both layers at the same time. The concept of cross-layer fingerprinting enhances security by demanding the simultaneous matching of multiple characteristics, yet it does not inherently provide protection against a determined attacker skilled in harvesting traits, acquiring identical hardware, or exploiting BLE pairing vulnerabilities. To ensure robust anti-spoofing measures, a dedicated threat model should be developed and evaluated experimentally, simulating adversaries who attempt to spoof link-layer and GATT-layer features simultaneously, in order to empirically quantify the system’s resistance to coordinated cross-layer impersonation rather than inferring it indirectly from identification accuracy. Additionally, expanding deployment could involve setting up lightweight edge-gateway configurations to enhance the efficiency of the real-time identification process.

## Figures and Tables

**Table 1:** Comparison of BLE device identification solutions.

Solution	Features Utilized	Core Methodology	No Retraining	Pre-Association Identification	Validated at Scale
BLENDER [12]	MAC addresses and one GATT service (DIS).	SHA-1 Hashing: Standard cryptographic hashing.	yes	No	Limited
MLP broadcast based solution [15]	Broadcast packets fields (omit scan response packet)	MLP classifier	No	yes	Limited (15 device type-manufacturers)
BF-IoT [14]	Flow-level traffic features: Link + flow (no GATT content)	Random Forest classifier	No	No	Limited (10 devices)
BleScope [16]	Static UUIDs harvested from companion mobile apps and BLE advertisement packets (ADV_IND, ADV_NONCONN_IND)	Static binary analysis (backward slicing + VSA on Android APKs); static UUID database matching	yes (but restricted to devices with published apps)	yes	yes (5K)
Celosia et al. [13]	gatt content	ATT entropy analysis (theoretical)	N/A	N/A	N/A
**Proposed Solution (Nilsimsa)**	**Cross-layer: Broadcast (ADV_IND + SCAN_RSP) & structured GATT (GAP, DIS, Custom)**	**Locality-Sensitive Hashing (LSH): Hashes raw byte sequences directly**	**yes**	**yes**	**yes (10K/5K)**

**Table 2:** Comprehensive BLE Feature Set for Device-Type Identification.

Category	Layer	Feature	Description	Example Value
Broadcast Packet	Link	ADV_IND header	PDU header defining standard undirected advertisement	0x1820
		ADV_IND length	Total byte count of the advertising payload	24
		SCAN_RSP header	PDU header identifying a scan response packet	0x2204
		SCAN_RSP length	Payload byte count of the scan response	34
		Advertising address	48-bit unique or randomized MAC address	38:39:8f:e9:c7:1f
		Scan response data	Raw hexadecimal payload of the scan response	0a:08:53:42:4d:4f:33:5a:10:ff:a9:0b
		CRC	24-bit checksum for packet integrity verification	0x252d9b
		Adv_IND entry	3 aggregated fields of one AdvData structure: length, type, and 16-bit service UUID	4, 0x16, 0xfe35
		SCAN_RSP entry 1	3 aggregated fields of one AdvData structure: length, type, and device name	29, 0x09, ShellyPlusPlugS-FCB46744FAD8
		SCAN_RSP entry 2	3 aggregated fields of one AdvData structure: length, type, and company ID	26, 0xff, 0x0ba9
Advertisement Pattern	Link	Event interval	Fixed time duration between consecutive advertising events	31 ms
		Channel sequence	Order of advertising channel transmission	(38, 39, 37)
		Advertising delay	Pseudo-random offset (0–10 ms) to prevent collisions	9 ms
GAP Service	GATT	Device Name (0x2A00)	Human-readable device identifier	SBMO-003Z
		Appearance (0x2A01)	Encoded category (phone, watch, plug, etc.)	motion detector, camera
		PPCP (0x2A04)	Min/max interval, latency, timeout parameters	14 00 28 00 00 00 E8 03
		Resolvable Private Address Only (0x2AC9)	RPA-only mode indicator	00/01
		Central Address Resolution (0x2AA6)	Address resolution capability indicator	00/01
GATT Service	GATT	Service Changed (0x2A05)	Notification of GATT database modifications	00 00 00 00
		Client Supported Features (0x2B29)	Bitfield of supported client features	00/01
		Database Hash (0x2B2A)	128-bit hash of the GATT table	25 e2 4f c8 2d 00 1e 2e 19 bb 5e 86 9b 76 ef e1
Device Information Service	GATT	Manufacturer Name (0x2A29)	Company that produced the device	Shelly, microsoft, govee
		Model Number (0x2A24)	Manufacturer-assigned model identifier	NRF52840-DK, Shelly Plus Plug S
		Serial Number (0x2A25)	Unique hardware unit identifier	SN-2024-78432-B
		Hardware Revision (0x2A27)	Physical circuit board version	Rev 1.2/Rev A
		Firmware Revision (0x2A26)	Embedded code version	20260914-090507/v1.14.0/1.2.3
		Software Revision (0x2A28)	Application software version	v2.1.0/2.0.1
		System ID (0x2A23)	64-bit identifier derived from MAC address	38 39 8f ff fe e9 c7 1f
		PnP ID (0x2A50)	Vendor ID, Product ID, and Version for driver matching	02 8a 24 66 82 01 00
		IEEE 11073 (0x2A2A)	Regulatory certification data	01 06 01 00 12 34 56 78
Custom Services	GATT	Manufacturer-defined services	Proprietary services with vendor-specific characteristics	de8a5aac-a99b-c315-0c80-60d4cbb51225

## Data Availability

The original contributions presented in this study are included in the article. Further inquiries can be directed to the corresponding author.
